# Nutritional Status and Nitrogen Uptake Dynamics of Waxy-Type Winter Wheat Under Liquid Organic Fertilization

**DOI:** 10.3390/plants14243799

**Published:** 2025-12-13

**Authors:** Aušra Arlauskienė, Danutė Petraitytė, Tadas Palubinskas, Marlo Jimenez, Jurgita Cesevičienė

**Affiliations:** 1 Institute of Agriculture, Lithuanian Research Centre for Agriculture and Forestry (LAMMC), Instituto Av. 1, Kėdainiai District, LT-58344 Akademija, Lithuania; 2 Idavang Ltd., A. Goštauto Str. 40 B, 8th Floor, LT-03163 Vilnius, Lithuania; 3 L’Institut Agro Dijon, Boulevard Dr. Petitjean, BP87999, 21079 Dijon, France

**Keywords:** fertilization strategy, gross energy, liquid anaerobic digestate, nitrogen rates, nitrogen use efficiency, low input wheat, pig slurry, soil mineral nitrogen, sustainable agriculture, waxy winter wheat

## Abstract

The growing demand for sustainable farming has increased interest in niche crops, including waxy wheat (*Triticum aestivum* L.). However, their nitrogen (N) nutrition characteristics from organic and mineral fertilizers are not sufficiently studied. In this research, the effects of pig slurry and liquid anaerobic digestate, as a sustainable alternative, were investigated and compared to ammonium nitrate, on waxy winter wheat, using N application rates of 0, 60, 120, and 120 + 50 kg ha^−1^ (the additional 50 kg ha^−1^ was applied as ammonium nitrate). The experiments were conducted in the northern part of Lithuania at the Joniškėlis Experimental Station of the Institute of Agriculture, Lithuanian Research Centre for Agriculture and Forestry (LAMMC) on clay loam *Cambisol* and repeated over two years (2019/2020 and 2020/2021) by reseeding winter wheat. The study evaluated mineral N in the 0–60 cm soil layer during active growing and autumn–non-vegetation periods, N accumulation in plant biomass, wheat grain and straw yield, fertilizer N use efficiency (NUE), and total energy yield. It was found that more than half of the total N required by the crop was taken up during the first half of the vegetation period (in favourable years—56%; in less favourable years—75% of the total required N). The optimal N rate for waxy winter wheat was 60–120 kg ha^−1^. The fertilizer’s NUE depended on the N rate; in favourable years, NUE values were 50–75% for N60, 19–43% for N120, and 29–40% for N120 + 50. Results indicate that biogas slurry can serve as a sustainable alternative for winter wheat main N fertilization, contributing to improved environmental outcomes.

## 1. Introduction

The growing global population, combined with the increasing demand for food production under changing climate conditions, has created an urgent need to conserve natural resources for sustainable agricultural practices [1]. To achieve the United Nations Sustainable Development Goals by 2030, it is essential to implement evidence-based, context-specific strategies that balance productivity, resilience, and environmental protection [2].

In this context, organic fertilizers—such as biogas anaerobic digestate (AD) and pig slurry (PS)—have gained attention as viable alternatives to synthetic fertilizers due to their potential to improve soil health, enhance crop productivity, and reduce environmental impacts [3].

Anaerobic digestate, derived from organic waste—such as animal manure and crop residues—through anaerobic digestion, serves as a nutrient-rich fertilizer that enhances agricultural sustainability and supports a climate-neutral economy in the agro-industrial sector [4,5,6].

A key difference between organic and conventional fertilizers lies in their nutrient composition. AD is a nutrient-rich fertilizer containing nitrogen, phosphorus, and potassium, which support plant health and soil fertility [7,8], making AD a valuable fertilizer. Unlike chemical fertilizers, which can lead to soil degradation and nutrient leaching, digestates contribute to the organic matter (OM) content of the soil, enhancing its structure and microbial activity [8]. AD exact composition depends on factors such as biogas plant operation, storage strategy, and feedstock type.

Studies have indicated that AD can perform as effectively as, or even better than, synthetic fertilizers in terms of crop yields, including wheat production [5]. Also, field trials on mixed pasture ley demonstrated that liquid digestate applications resulted in grass yields comparable to those achieved with conventional inorganic fertilizers [7]. The repeated application of AD has shown the ability to enhance soil quality over time. Unlike chemical fertilizers, which can contribute to soil degradation and nutrient leaching, AD improves soil OM content, structural integrity, and microbial activity [8]. Studies indicate that long-term use of AD can enhance soil fertility and reduce reliance on synthetic fertilizers [7,9].

Anaerobic digestate and pig slurry exhibit different effects on soil properties. The anaerobic digestion process increases the stability and nutrient concentration of AD compared to raw PS by reducing OM content and altering nutrient distribution, making nutrients more accessible to plants [10]. While PS may provide more rapid OM decomposition, AD decomposes at a lower rate (about 3% of added total organic carbon). PS decomposes more rapidly (25 to 50%), which affects nutrient cycling and microbial activity [10]. The anaerobic digestion process reduces the volatile fatty acid content and other volatile organic compounds, resulting in a higher quality more biologically stable product with improved fertilizing potential than the initial feedstock mixture [11].

Choosing between AD and PS requires consideration of specific soil and crop needs, along with environmental factors such as heavy metal content and emission potential. Some studies indicate that AD may present environmental and human health risks due to higher ammonia emissions and potential heavy metal contamination compared to undigested animal manures and slurries [12,13,14]. Therefore, careful management practices are essential to maximize benefits while minimizing risks.

Common winter wheat and waxy winter wheat (both *Triticum aestivum* L.) differ significantly in their nutritional composition and technological properties, with starch composition being the primary distinction. Common wheat contains both amylose and amylopectin starch, while waxy wheat has very low amylose content and is characterized by its high amylopectin content. This unique starch profile provides valuable functional properties for food processing, baking (enhances the quality and shelf life of baked and frozen products, reduces cooking time), and industrial applications (including adhesives, textiles, paper, and ethanol production), and has led to increasing interest in waxy wheat in recent years [15,16,17,18].

However, waxy wheat has distinctive properties but generally produces lower yields than common winter wheat due to reduced sucrose-to-starch conversion in the late grain filling stage, resulting in lower kernel weight and decreased total starch accumulation [19] with reported yield reductions of 20–24% [20] and lower nitrogen use efficiency (NUE) [18]. For example, the proportion of nitrogen (N) fertilizer used for grain production shows that the waxy cultivar ‘Sarta’ was more stable than other cultivars but still utilized only 51–56% of applied N for grain yield, whereas other cultivars used 60–87% [18]. Existing studies mainly focus on grain quality and starch characteristics, whereas information on soil responses and nutrient dynamics during waxy wheat cultivation is largely absent [18,19,20]. Limited cultivars more adapted to European environments and scarce data on N management further constrain its productivity, highlighting the need to understand its specific N demand and uptake patterns.

As a staple crop grown worldwide, wheat production relies heavily on the efficient uptake and utilization of N [21]. Nitrogen is a key macronutrient that influences plant growth, biomass accumulation, and grain protein content [22,23,24]. Although large amounts of N fertilizers are used worldwide, the efficiency of N fertilizers is relatively low—ranging from 25 to 50% of the applied N [25,26,27]. However, inefficient N use can contribute to environmental pollution, soil degradation, and economic losses for farmers. Therefore, understanding N uptake dynamics in winter wheat (also including and waxy wheat) under varying nutritional conditions is essential for optimizing fertilization efficiency.

The dynamic of soil N in winter wheat cultivation is strongly affected by meteorological conditions, especially precipitation and temperature. Its content fluctuates based on farming practices and environmental factors [28]. High precipitation reduces soil water storage at anthesis and maturity as N application rates increase, impacting N availability [29]. Additionally, seasonal changes in soil respiration, closely tied to N cycling, are strongly influenced by soil temperature [30]. Drought conditions can slow straw decomposition, resulting in higher residual mineral N in the soil and reduced plant N uptake. In contrast, increased rainfall accelerates decomposition, promoting greater N mineralization and availability [31].

Anaerobic digestate and pig slurry were selected because they represent the two most widespread liquid fertilizers available to farmers in livestock-intensive and biogas-producing regions. Pig slurry is a primary by-product of intensive pig farming, rich in ammonium nitrogen and OM. Anaerobic digestate is produced through the microbial decomposition of organic substrates under oxygen-free conditions in biogas reactors, a process that converts part of the OM into methane and CO_2_ and results in a fertilizer with a relatively low C/N ratio and high ammonium content. The composition of PS and AD, including their nutrient content and OM composition, can vary depending on factors such as the animals’ feed, pig housing, and rearing practices, as well as the part and type of organic waste used for biogas/digestate production [12]. It is important to consider appropriate application rates for these fertilizers to maximize their benefits and minimize potential negative environmental impacts.

This research is in line with actualities, as the future of agriculture will increasingly depend on the use of liquid organic fertilizers derived from agricultural and agro-industrial waste streams, enabling the production of environmentally friendly products, increasing production volume, and reducing ecological impact. Additionally, biogas AD and PS are alternatives to synthetic fertilizers, promoting sustainable agriculture through the recycling of agricultural waste, lowering mineral fertilizer usage, and protecting the environment.

Given these considerations, we hypothesized that the use of treated (digestate) and untreated (pig slurry) liquid organic fertilizers for fertilization will have a positive effect on nitrogen uptake in low-intensity waxy winter wheat.

The objective of this study was to compare the effects of liquid anaerobic digestate, pig slurry, and ammonium nitrate fertilizers on nitrogen accumulation, utilization efficiency, and gross energy yield of waxy winter wheat (*Triticum aestivum* L.).

The findings of this study will contribute to a better understanding of the advantages of recycling organic waste (AD) within the production cycle, the mechanisms of nitrogen uptake in waxy winter wheat, and the benefits and limitations associated with the use of organic fertilizers. Moreover, the study will provide insights into sustainable technologies for their application. The results are expected to be relevant not only for the cultivation of waxy winter wheat but also for common soft winter wheat.

## 2. Results

### 2.1. Variation in Soil Nitrogen Supply Due to the Application of Organic Fertilizers During Two Years of Winter Wheat Production

Soil mineral N traits were analyzed using a three-way analysis of variance (ANOVA). The results showed that in the 0–60 cm soil layer, mineral N concentration and its variations were significantly influenced by the interaction between sampling time and N rates, as well as sampling time and fertilizer type (*p* < 0.01). In the first year of the experiment, mineral N levels in unfertilized (with N) waxy winter wheat plots consistently increased throughout the vegetation period, and the highest levels were observed after harvesting (Table 1). During the autumn–winter period, mineral N content decreased by 9.15 mg kg^−1^ soil or 31.0%. In the next year, when wheat was grown in a repeated cropping system, mineral N concentrations increased only slightly during intensive growth. However, in the second half of the growing season, mineral N levels declined by 2.3 mg kg^−1^ or 50.9%. The data in unfertilized plots showed the influence of the interaction between soil and meteorological conditions on soil N supply to wheat.

The trends in mineral N variation in fertilized plots remained consistent; however, fertilizer N rates played a significant role. In the first year of intensive wheat growth, N fertilization increased mineral N content by an average of 22.0% compared to unfertilized plots. During the first half of the wheat growing season, mineral N increased by 6.20–6.67 mg kg^−1^, with no significant differences between fertilizer rates (Table 2. The differences in periods are calculated from Table 1). After wheat harvest, N rates of 120 and 120 + 50 kg ha^−1^ resulted in significantly higher mineral N contents (3.82 and 3.77 mg kg^−1^, respectively) compared to the intensive growth period, while the 60 kg N ha^−1^ fertilizer rate had the lowest soil mineral N content.

During the autumn–winter period, all plots under repeated wheat cultivation experienced mineral N losses of 8.16–11.03 mg kg^−1^, decreasing 2.75–3.44 times (the differences were significant) compared to post-harvest levels (Table 2). No significant differences between N rates were observed after wheat resumed vegetation (2AV) or during its intensive growth phase (2IG). In the first half of the growing season, mineral N content increased by 31.2–45.5% compared to the unfertilized (N0) plots, with the highest increase at 120 kg N ha^−1^ rate. After the winter wheat harvest (2AH), fertilization with 60 and 120 kg N ha^−1^ significantly reduced mineral N content by 47.6 and 48.0%, respectively, compared to the 2IG period. The highest mineral N levels were recorded with the 120 + 50 kg N ha^−1^ treatment, showing a significant difference from other rates.

The mineral N content of the soil was significantly affected by the types of fertilizer (Table 1 and Table 2). In the first year of wheat cultivation (1IG), the mineral N content was significantly higher with anaerobic digestate (AD) compared to ammonium nitrate (AN) or pig slurry (PS), with increases of 31.3 and 24.2%, respectively. Compared to the start of the growing season, AD increased the mineral N content by 8.49 mg kg^−1^, or 2.7 times. There were no differences between AN and PS.

After crop harvest, no significant differences in mineral N content were observed between fertilizer types, though AD tended to increase the content. During the autumn–winter period (2AV–1AH), mineral N losses after application of the different fertilizers were similar. However, AD contributed to the highest increase in losses (10.55 mg kg^−1^). At the resumption of winter wheat vegetation, no significant effects were observed, although liquid organic fertilizers tended to increase mineral N content.

During the period of intensive wheat growth (2IG), significantly higher mineral N content was found in the plots fertilized with ammonium nitrate (AN), where mineral N increased by 79.6% (compared to 2AV). In contrast, liquid organic fertilizers resulted in a 23.3–32.3% increase. These differences were statistically significant. After harvest, the soil mineral N content was similar across plots with different fertilizer types. In the second half of the growing season, AN was the most effective in utilizing mineral N, likely due to better N uptake. Over two years of winter wheat cultivation with the same fertilization, no differences between fertilizer types were observed.

### 2.2. Nitrogen Use by Waxy Winter Wheat During the Period of Intensive Growth

Unfertilized waxy winter wheat showed the highest dry matter (DM) accumulation during the period of intensive growth and development of the above-ground mass (Table 3). Fertilized crops exhibited slower development and DM accumulation. The lowest DM accumulation was observed in the biomass of crops fertilized with PS120, where the highest change in DM (9.6%) compared to the control was also noted. However, the total above-ground mass was inversely proportional to DM accumulation.

Compared to the control, a significant increase in the yield of above-ground mass was observed in crops fertilized with the 120 kg ha^−1^ N rate, regardless of the fertilizer type. Although the variation was similar between treatments, it was not statistically significant. Wheat in repeated cultivation accumulated, on average, 16.6% less DM under unfavourable growing conditions in 2021 than in the favourable 2020 season. The lower DM accumulation in biomass and its higher variation was attributed to AN fertilization (irrespective of N rate).

In terms of above-ground mass, higher DM accumulation was achieved with liquid organic fertilizers, with AD showing the most consistent results. PS was more effective at a low N rate (N60). The highest above-ground mass of wheat was observed with the 120 kg ha^−1^ N rate fertilization, showing significant differences compared to the control. The significantly greater change in above-ground mass compared to the control was recorded with AN120 fertilization.

All fertilizers and N rates (except PS60) resulted in a significant increase in the N concentration of winter wheat above-ground mass (Table 4). Fertilization significantly increased N accumulation in wheat biomass, with an average increase of 44.6% compared to unfertilized wheat. The highest N content and the greatest N increment due to fertilization were found in the biomass of wheat fertilized with PS120. The highest N use efficiency (NUE) was observed at the lower N fertilization rate (60 kg ha^−1^), with efficiency decreasing as the N rate increased. No consistent trends were observed between fertilizer types. When waxy wheat was grown as a repeated crop (2021), the average biomass N concentration was 7.7% higher compared to 2020. Only AN significantly increased the biomass N concentration. However, wheat biomass N accumulation was 25.9% lower, on average, compared to 2020. The highest biomass N content and the greatest N increase were observed with AN120 fertilization. Liquid organic fertilizers (PS, AD) applied at 120 kg ha^−1^ N rate also significantly increased N accumulation in the biomass. In the less favourable year for winter wheat intensive growth, the highest NUE was found with AS fertilization (N60—18.8%, N120—19.9%).

### 2.3. Nitrogen Accumulation in Waxy Winter Wheat Yield and Nitrogen Uptake Intensity Throughout the Growing Season

**Figure 1:**
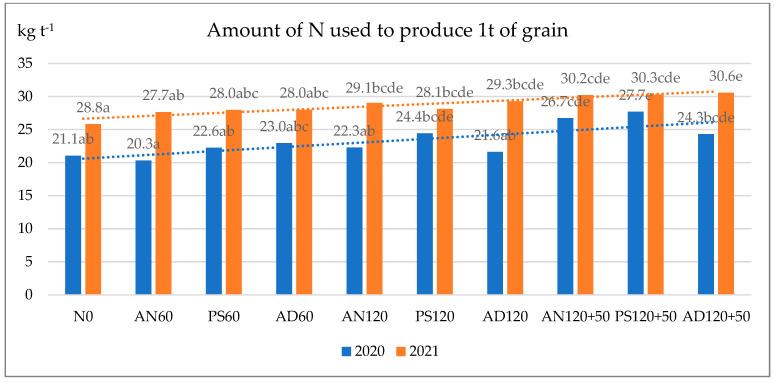
Nitrogen inputs per tonne of waxy winter wheat (*Triticum aestivum* L.) grain yield (BBCH 87). Fertilizers: AN—ammonium nitrate, PS—pig slurry, AD—anaerobic digestate. Nitrogen (N) rates: N0—unfertilized; N60—60 kg N·ha^−1^; N120—120 kg N·ha^−1^; N120 + 50—120 + 50 kg N·ha^−1^. The additional N fertilization (+50) was always applied as ammonium nitrate. Data with different letters are significantly different at the *p* < 0.05 level in separate years.

The N use intensity data (BBCH 87) for the entire growing period of waxy winter wheat is presented in Table 5 and Figure 1. In 2020, an average increase in the grain N concentration was 6.5% at 60 kg ha^−1^, 10.1% at 120 kg ha^−1^, and 25.4% at 120 + 50 kg ha^−1^ compared to unfertilized crops (Table 5). The most consistent increase in N concentration was observed in plots with PS, showing a significant increase at medium (N120) and higher (N120 + 50) rates. In the less favourable year for waxy wheat growth (2021), N concentration was, on average, 27.8% higher than in the favourable 2020 season. As fertilizer rates increased, grain N concentration also significantly increased (except AN60). Lower and medium N rates resulted in a 6.5–12.0% increase in grain N concentration, with no significant differences between these rates. However, when fertilized at a higher N rate over two applications, the average increase in grain N concentration was 16.1%. In 2021, the highest grain N concentrations were recorded with PS120 + 50 and AD120 + 50 fertilization.

In 2020, lower and medium N fertilizer rates resulted in similar N accumulation in winter wheat (grain + straw) yield. Compared to the control, N accumulation increased by an average of 55.8% with these treatments. While at the highest N fertilizer rate, accumulation increased by an average of 85.5%. Pig slurry (PS120, PS120 + 50) resulted in significantly higher N accumulation in the yield. In contrast, in 2021, N accumulation in the winter wheat crop was moderate, reaching 59.56 kg ha^−1^—44.3% less than in 2020. Fertilization significantly increased N accumulation in the crop (except AD60). Fertilization at a rate of 60 kg N ha^−1^ led to an average N increase of 37.4%. Increasing the N rate resulted in a gradual rise in N accumulation in the yield, with 64.8% at 120 kg ha^−1^ and 73.8% at 120 + 50 kg ha^−1^. The highest N accumulation in the yield was observed with ammonium nitrate (AN120 and AN120 + 50).

Regarding fertilizer NUE, the highest N uptake (49.5–74.9%) was recorded at the lowest N rate (60 kg ha^−1^). However, fertilizer efficiency decreased as N rates increased. Comparisons between fertilizers showed that plants absorbed more N from liquid organic fertilizers (especially PS) than from ammonium nitrate. In the second year, fertilizer NUE was lower (15.0–30.2%), with ammonium nitrate showing the highest N efficiency.

The N input per tonne of grain production (Figure 1) showed that, in the favourable 2020 season, similar amounts of N (20.3–24.4 kg t^−1^) were used per tonne of grain in unfertilized and lower-to-medium fertilized crops, with no significant differences from the control. Additional fertilization (N120 + 50) led to an average increase of 24.6% in N applied per tonne of grain. The highest N inputs were observed with ammonium nitrate and pig slurry (AN120 + 50 and PS120 + 50), with significant differences compared to the control. In the less favourable 2021 season, N inputs increased by an average of 22.6% compared to 2020. Fertilizing wheat at medium or higher N rates significantly increased N inputs (by 11.8 and 17.7%, respectively). However, no significant differences were observed between the medium and higher N rates or between the different fertilizer types.

### 2.4. Productivity of Waxy Winter Wheat

The winter wheat grain yield accumulated different amounts of gross energy (GE), averaging 84.1 GJ ha^−1^ in 2020 and 38.5 GJ ha^−1^ in 2021 (Table 6). This highlights the stark contrast between the two years, with meteorological conditions significantly influencing crop growth, development, and productivity. Compared to the control, fertilization led to significant differences in gross energy content. However, increasing N rates was not equally effective each year.

In the first year of waxy winter wheat cultivation (2020), the most effective N rate was 60 kg ha^−1^, resulting in a 50.7% increase in gross energy. Fertilization at medium and higher N rates led to only slight variations in gross energy content. When repeated cultivation winter wheat was grown under unfavourable conditions in 2021, gross energy accumulation was low. While the lower N rate increased gross energy content by 28.2% on average, the medium and higher rates (120 kg N ha^−1^ and 120 + 50 kg N ha^−1^) were more effective, leading to an average increase of 48.4% compared to the control. In an unfavourable year for wheat growth, the most significant increase in gross energy was observed with ammonium nitrate (AN) applied at medium and higher N rates. Over the two years combined, the lower N rate increased gross energy by 43.4%, the medium rate by 43.9%, and the higher rate by 48.0% compared to the control (the difference was significant). Slow-acting liquid organic fertilizers increased gross energy content more (PS—49.2%, AD—44.1%) than AN (42.0%), though the differences were not statistically significant.

## 3. Discussion

In modern agriculture, N remains the key nutrient limiting the yield of most crops. However, the use and efficiency of N fertilizers are associated with significant losses [32]. Typically, N fertilizers operate within a complex interaction of soil properties and climatic conditions. Nitrogen accumulation in winter wheat biomass occurs unevenly throughout the growth period. The most intensive N uptake occurred between mid-April and mid-June, aligning with rapid plant development and DM accumulation. Up to 80% of the total N is absorbed by the flowering stage in mid-June [33]. According to Cameron et al. [34], up to 40% of applied fertilizer N is taken by plants within a relatively short period of 30 days. Other studies report that, depending on the type of digestate used, wheat can achieve 58.9–74.2% N offtake variance [35].

Based on our research, by mid-May, winter wheat in aboveground biomass had accumulated on average 56.4 and 75.1% of the total N absorbed during the growing season (Table 4 and Table 5). Mineral N fertilizers (AN) were more effective, especially in 2020. In that year, pig slurry (PS) resulted in higher N accumulation in the yield compared to anaerobic digestate (AD). Nitrogen uptake in 2020 may have been reduced by hot and dry weather conditions in July, which accelerated crop maturation. In spring 2021, low N accumulation in aboveground biomass was limited by moisture deficiency during April and May. Additional fertilization during the second half of the growing season did not compensate for the unabsorbed N content. It has been stated that intensive N accumulation from germination to flowering results in more efficient fertilizer use, which is the basis for high yields [36]. Therefore, the main N fertilization of winter crops at the beginning of growth resumption is crucial.

Nitrogen fertilizers application enhances aboveground DM and grain N accumulation at maturity [37]. Nitrogen use efficiency (NUE) in winter wheat depends on the N input level and meteorological conditions, influencing yield and energy production. Yield increases usually slows down when the annual N application exceeds 50 kg N ha^−1^. Excessive N use can decrease NUE and negatively affect yield [37,38,39,40]. This inverse relationship highlights the need for optimized fertilization to improve efficiency. In our study, the highest NUE was observed at an application rate of 60 kg N ha^−1^. Pig slurry was more efficient than anaerobic digestate (Table 5). On average, fertilizer efficiency was relatively low—42.8% in 2020 and 20.8% in 2021. The tendency for high N loss reduces fertilizer efficiency [31]. Meteorological conditions play a crucial role in N inputs and the energy yield of winter wheat, emphasizing their complex interactions. Optimal N application rates for winter wheat depend on various environmental factors [40,41]. Future climate change, involving shifts in temperature and precipitation, is projected to reduce winter wheat yields by 2~4% and decrease total N losses by up to 5%. But, in some regions, total N losses may rise by up to 5% and N leaching per unit yield may increase by 0~10% [41]. However, using enhanced-efficiency fertilizers and optimizing application timing can help mitigate climate change impacts and improve NUE [42].

Digestate, as an organic fertilizer, has a specific N composition and behaviour. The chemical composition and fertilizing value of digestate largely depend on the type of substrates used during anaerobic digestion and on technological conditions [35,43]. It has been found that the N-NH_4_/N ratio in digestate from different biogas plants may vary; however, these differences usually have only a limited impact on agronomic efficiency after incorporation into soil [34]. One of the main characteristics of digestate is its high ammonium nitrogen (N-NH_4_) concentration, which is directly available to plants—this distinguishes it from other organic fertilizers. Nevertheless, the N efficiency of digestate is often lower than expected.

Digestates typically have a low carbon-to-nitrogen (C/N) ratio, since a large portion of OM is metabolized into methane and carbon dioxide during anaerobic digestion. When digestates are applied to soil, microorganisms can immobilize N by utilizing available organic carbon as an energy source, thereby slowing N availability to plants [44,45]. According to Cao et al. [46], not only the C/N ratio but also the form of organic carbon compounds—mainly labile compounds—can promote microbial N immobilization. However, microorganisms immobilize a small portion of mineral N, and most of it remains available to plants [47,48]. Depending on digestate type, immobilization may last 20–40 days after application, followed by remineralization when N again becomes available to plants [45]. Fertilizer chemical analyses showed that the composition of liquid organic fertilizers varied between types and years. Pig slurry (PS) contained more organic matter (OM) and organic carbon (C_org_) than anaerobic digestate (AD), indicating a higher potential for microbial activity and temporary N immobilization. Meanwhile, AD had a lower C/N ratio—especially in 2021 (1.87)—which favours faster N mineralization. Interannual variation was also evident: in 2020, AD contained more C_org_ and NH_4_-N, which could have slowed its initial N release, whereas PS in 2021 had higher OM and total N, what can potentially enhance its fertilizing effect. Overall, these chemical variations help explain the observed trend of higher soil mineral N concentrations (2020) after AD application (compared to PS), both during intensive growth and after harvest (Table 1), indicating higher N availability in later stages. The late incorporation of digestate may even reduce its N fertilizer value, due to stronger N immobilization relative to NH_3_ emissions [35]. Comparisons between the use of plant residues and biogas digestate indicates that biogas digestion can create a win–win scenario, providing additional energy yields while simultaneously reducing nitrate leaching risk and nitrous oxide emissions [49]. Nevertheless, environmental risks associated with digestate have also been reported due to potentially higher NH_3_ emissions compared to undigested OM [49,50]. On the other hand, due to the active organic carbon contained in digestates, they can provide greater benefits for organic soil matter than the original raw material [48]. The mineralization of organic N to NH_4_ increases the ammonium fraction and improves direct N availability [51]. Some studies indicate that nitrification of NH_4_ occurs more slowly in digestate-treated soils compared with mineral fertilizers [50], which can also affect plant N uptake.

It is important to note that digestate application should follow the same agronomic principles as traditional liquid fertilizers. As reported by Abebe and Feyisa [52], low fertilizer efficiency is often related to poor synchronization between the timing of N application and crop demand. The time lag between N application and active uptake allows for N losses via leaching, clay fixation, immobilization, and denitrification [52,53].

Low fertilizer efficiency is often associated with N losses due to NO_3_ leaching, NH_3_ volatilization, surface runoff, and denitrification [32]. Other researchers indicate that NH_4_ volatilization between 0 and 40 days after application cannot be completely excluded, even with precautionary measures [45]. According to Tiwary et al. [54], these losses can reach 35–65% of total applied N if digestate is not incorporated into the soil. Emissions depend on both feedstock and soil characteristics, such as OM content, texture, water content, and aeration [47,55].

Regional climate—including temperature and precipitation—also affects N availability, residue decomposition, and organic matter mineralization rates. Field soils also have various properties (texture, pH, and OM content), that influence N losses by promoting leaching or denitrification in wetter years or affecting mineralization dynamics [34]. The amount of stress relief can be controlled by the type of N, either ammonium (NH_4_) or nitrate (NO_3_). N-NO_3_ may exhibit greater tolerance to heat stress than N-NH_4_ [56].

Overall, AD application may enhance microbial activity and biomass [57], N mineralization, and NH_3_ oxidation [58], as well as the mineral N content of soils [59]. Soils with a pH above 6.0 tend to increase NH_3_ losses. When assessing AD performance, it is important to consider not only N but also phosphorus and potassium [60].

To reduce NH_3_ volatilization, organic fertilizers including aerobic digestates should be incorporated as soon as possible [54,59] and applied through injection [61], strip spreading [62], acidification [63], or the use of nitrification inhibitors [59,64]. Due to N immobilization, appropriate timing of fertilization is crucial to avoid immobilization and negative effects on crop growth [35,45].

The benefits of AD depend mainly on soil properties rather than the amount or quality of OM applied. In soils with a neutral pH, the highest levels of OM, microbial biomass, fungi, bacteria, and cation exchange capacity were observed—these parameters increased over time in both solid and liquid fractions [65]. Researchers have reported that digestate application enhances crop yield and improves N and P uptake efficiency [64,66].

In summary, digestate has strong potential as a valuable N fertilizer; however, its efficiency depends on multiple factors, including the chemical composition, C/N ratio, substrate origin, application technology, and timing. Achieving high agronomic efficiency requires careful consideration of fertilization timing, soil characteristics, crop demand, and potential microbial N immobilization.

## 4. Materials and Methods

### 4.1. Experimental Site and Soil

The studies were carried out from 2019 to 2021 at the Joniškėlis Experimental Station of the Institute of Agriculture, LAMMC (56° 210′ N, 24° 100′ E) on drained heavy loam on silty clay, with a deeper sandy light loam soil and limnoglacial clay on moraine loam as the parent material. According to the Lithuanian soil classification, the soil is classified as a deeper carbonate deeper gleyic Cambisol (Rdg4-k2) *Endocalcari Endohypogleyic Cambisol* (Cmg-n-w-can). The topsoil contains 27% clay particles (<0.002 mm), while the subsoil is a clay-rich layer with more than 50% clay particles. Limnoglacial sediments, about 45 cm thick, are sandy light loam, while the bottom moraine layer is deeper than 70–80 cm, and has carbonates starting at 50–60 cm depth. At the time of experimental installation, the topsoil (0–30 cm) was close to neutral (pH_KCl_ 6.5), moderately phosphatic (P_2_O_5_ 113 mg kg^−1^), potassium-rich (K_2_O 267 mg kg^−1^), moderately humified (22.9 g kg^−1^) and had a moderate total N content (N_tot_ 1.13 g kg^−1^).

The initial plot size was 20 × 5 m = 100 m^2^, and the experimental plot size was 15 × 2.2 m = 33 m^2^, with 3 replicates. The plots in the winter wheat strip were arranged in a randomized block design.

### 4.2. Experimental Design and Details

The amylopectin starch-synthesizing waxy winter wheat (*Triticum aestivum* L.), variety *Eldija* (amylose content close to 0%), was cultivated for two consecutive years (2019–2020 and 2020–2021) at the same site. The seed rate used was 4.5 million germinated seeds per ha. The pre-crop grown before the experiment was winter wheat.

Liquid organic (PS and AD) and granulated mineral (AN) fertilizers were surface applied manually by simulating machinery fertilization according to the scheme presented in Table 7, using the same plots in both years. All liquid organic fertilizers were supplemented with the nitrification inhibitor DMPP (3,4-dimethylpyrazole phosphate), used as a commercial product Vizura (BASF) at a rate of 2 L ha^−1^. The inhibitor was mixed into the liquid organic fertilizers immediately prior to their application at the winter wheat regrowth stage (BBCH 25). According to the product application guidelines, the nitrification inhibitor suppresses the activity of *Nitrosomonas* bacteria in the soil. As a result, ammonium nitrogen cud remains in the NH_4_^+^ form for longer period in soil particles instead of being rapidly converted into nitrate and potentially lost through leaching.

Winter wheat main N fertilization was caried out after the resumption of spring vegetation on 24 March 2020 and on 4 April 2021 at the BBCH 25 growth stage. Additional fertilization was conducted at the end of booting, during flag leaf formation (BBCH 35, N 50 kg ha^−1^), using ammonium nitrate (Table 7).

The characteristics of the main parameters of the liquid organic fertilizers are provided in Table 8. Overall, PS showed higher OM and C_org_ values compared to AD, while variations in N forms and C/N ratios reflect differences in composition between the two fertilizer types and between years.

The pig slurry (PS) was obtained from a pig farm (located at 55°42′54.8″ N, 23°46′33.4″ E, in Kauleliškiai, Radviliškis district). Liquid anaerobic digestate (AD) was obtained after biodigestion in a biogas plant (located at 56°03′54.6″ N 23°59′13.9″ E, in Veselkiškiai, Pakruojis district), where the primary feedstock consisted of pig slurry and industrial crop residues such as molasses, mill residues, starch feedstocks, cellulose, and sugar beet cake. A screw press separator was used in the biogas plant for solid–liquid separation of digestates prior to the post-fermentation storage in the lagoons. Both liquid fertilizers (~2 L) were sampled from the storage lagoons every spring before the main fertilization. Organic and mineral fertilizer rates were calculated on the base of the N concentration in the fertilizer (Table 8).

Every year, wheat straw was chopped and spread during the wheat harvest, and the stubble was chopped after harvesting using a universal stubble cultivator at a depth of 10–12 cm. Two weeks later, the stubble was ploughed at a depth of 25 cm. The soil was prepared for sowing with a combined pre-sowing cultivator. Before sowing, background fertilization with N_32_P_32_K_32_ complex mineral fertilizer was applied in both experimental years. The fertilizer rate was selected after assessing the plant-available phosphorus and potassium contents in the soil. The winter wheat was cultivated using intensive technology, which included seed treatment, one application of herbicides, two applications of fungicides, and one application of a growth regulator each year.

### 4.3. Meteorological Conditions

Meteorological conditions significantly influenced the growth and yield of winter wheat each year. Table 9 presents key meteorological indicators characterizing the 2019–2020 and 2020–2021 growing seasons. These seasons differed in terms of temperature patterns and precipitation distribution, which directly impacted crop development.

Growing season 2019–2020. September 2019 was dry, but sufficient rainfall in the first half of October supported wheat germination and autumn emergence. The winter was unusually warm, with average daily temperatures above 0 °C in all winter months. In March, the variation in daily minimum and maximum temperatures was large but did not lead to an earlier resumption of winter wheat vegetation. April was characterized by low rainfall, while May received optimum rainfall. Cooler weather in April and May, compared to the standard climatic norm (SCN), prolonged crop tillering. However, June and July were warmer and wetter than the SCN. Heavy rainfall in late June (29th—27.0 mm) and in July (9th—20.0 mm, 19th—24.0 mm) may have caused the lodging of more fertilized (N120 + 50) crops. In summary, the growing season was favourable for the growth of winter wheat.

Growing season 2020–2021. Late summer and early autumn of 2020 (August and September) were dry, resulting in poor germination of winter crops. Excessive rainfall in October combined with an unusually warm November, may have contributed to mineral N losses. The winter conditions were close to the SCN. When the growing season resumed in 2021, conditions were unfavourable for winter wheat growth and development due to an excessively dry April and an excessively wet May, dominated by torrential rainfall (41.5 mm on 3rd and 23.5 mm on 26 May). In June, total rainfall aligned with the SCN, but most of it fell in a single day (56.5 mm on 24 June). The dry and hot July shortened the growing season and accelerated crop maturity. In summary, this growing season was less favourable for winter wheat growth due to alternating dry periods and recurrent torrential rainfalls.

### 4.4. Soil, Organic Fertilizers, and Plant Analysis

The sampling and methods used to determine the soil background and composition of liquid organic fertilizers are provided in [67].

Soil samples during the experiment were collected at different stages: during vegetation recovery (AV) (as background samples from control plots in the first year and from all plots in the subsequent year), 5–6 weeks after the main application of organic and mineral nitrogen fertilizers (IG) (from both control and fertilized plots), and again after harvest (AH) (from all plots). Sampling dates were as follows: AV—24 March 2020 and 31 March 2021; IG—14 May 2020 and 20 May 2021; AH—12 August 2020 and 10 August 2021. At each sampling time, soil was taken from the 0–30 and 30–60 cm soil layers of each plot. Two composite samples were prepared per plot (one per layer), each consisting of five auger cores. For the determination of nitrate (N-NO_3_) and ammonium (N-NH_4_) nitrogen concentrations, the samples were crushed, frozen, and later combined in equal proportions into a single mixed sample representing the 0–60 cm soil layer. The article presents the calculated mineral nitrogen concentrations in this 0–60 cm soil layer. Changes in mineral nitrogen concentration during the growing season of winter wheat were calculated for three periods: (i) the first half of the growing season (1IG—1AV and 2IG—2AV), (ii) the second half of the growing season (1AH—1IG and 2AH—2IG), and (iii) the autumn–winter period (2AV—1AH).

Soil nitrate (N-NO_3_) nitrogen concentrations were determined using the potentiometric method (CyberScan 2100, Eutech Instruments, Vernon Hills, IL, USA) in a 1% extract of KAl(SO_4_)_2_ × 12H_2_O (1:2.5, *w*/*v*). Ammonium (N-NH_4_) nitrogen concentrations were measured using a spectrophotometric method (UV/Vis Cary 50, Varian Inc., Palo Alto, CA, USA) at a wavelength of 655 nm in a 1M KCl extract (1:2.5, *w*/*v*). Soil mineral nitrogen content was calculated by summing the concentrations of N-NH_4_ and N-NO_3_.

Plant samples were taken during the period of intensive crop growth (BBCH 35), before additional fertilization with ammonium nitrate (+N50), to determine the above-ground mass and N concentration of winter wheat. The crops were cut at the soil surface in two 0.5 m long rows in each plot. Winter wheat straw yield was determined by collecting 4 sheaves from 4 random spots (0.25 m^2^ each) in the pre-harvest plots. Grain yield was harvested and measured when most of the crops reached the hard dough stage (BBCH 87). Each experimental plot was harvested using a small plot combine harvester. Grain samples (1 kg) were taken from each plot. All plant-based samples were prepared to determine dry matter (DM) and nitrogen (N) concentrations. DM was determined by drying to a constant weight at 105 °C in a forced-air oven. Dried samples (at 60 °C) were ground using an ultracentrifugal mill ZM 200 (Retch, Haan, Germany) equipped with a 1 mm mesh size sieve. Whole plant, straw, and grain N concentrations were determined from sulfuric acid digestates using the Kjeldahl method with spectrophotometric detection at 655 nm after reaction with salicylate and hypochlorite ions (UV/Vis Cary 50, Varian Inc., Palo Alto, CA, USA). All concentrations of soil and plant N compounds were expressed on a DM basis. All chemical analyses were conducted at the Chemical Research Laboratory of the Institute of Agriculture, LAMMC.

The total nitrogen (N) accumulated in the above-ground biomass of winter wheat at the intensive growth (IG) stage ($N_{B}$, kg ha^−1^) and in grain and straw at harvest ($N_{G+S}$, kg ha^−1^) was calculated using the following formulas:

(1) $$N_{B}=\frac{N_{BC}\times Y_{B}}{100}$$

(2) $$N_{G+S}=\frac{N_{GC}\times Y_{G}}{100}+\frac{N_{SC}\times Y_{S}}{100}$$

where $N_{BC}$, $N_{GC}$, and $N_{SC}$ are the N concentrations in biomass, grain, and straw (% on a DM basis), and $Y_{B}$, $Y_{G}$, and $Y_{S}$ are the corresponding yields in DM (kg ha^−1^).

Fertilizer N use efficiency (NUE, %) in above-ground biomass at the IG stage or in grain and straw at harvest was calculated as

(3) $$\mathrm{NUE}=\frac{\left(N_{B}^{\mathrm{fertilized}}-N_{B}^{\mathrm{control}}\right)\times 100}{N_{\mathrm{rate}}}$$

(4) $$\mathrm{NUE}=\frac{\left(N_{G+S}^{\mathrm{fertilized}}-N_{G+S}^{\mathrm{control}}\right)\times 100}{N_{\mathrm{rate}}}$$

where ($N_{\mathrm{fertilized}}-N_{\mathrm{control}}$) is the difference in N accumulation between fertilized and unfertilized plants (kg ha^−1^), and $N_{\mathrm{rate}}$ is the N fertilizer rate (60, 120, or 120 + 50 kg ha^−1^). The calculations were not adjusted for background mineral N; however, the amount of N taken up by the plants in the control (unfertilized) treatment was taken into account.

The annual nitrogen input ($C_{N}$, kg t^−1^ of grain production) was calculated using the following formula:

(5) $$C_{N}=\frac{1000\times N_{G+S}}{Y_{G}}$$

Winter wheat grain yields were converted into energy units using generalized data on the energy value of crops grown in Lithuania [68]. Gross energy (GE, GJ ha^−1^) was calculated as

(6) $$GE=\frac{Y_{G}\times 18.56}{1000}$$

where 18.56 MJ kg^−1^ is the gross energy content of grain dry matter, and 1000 is the conversion factor from MJ to GJ.

### 4.5. Statistical Analysis

The SELEKCIJA software package [69] was used for statistical analysis of the data. Soil data were analyzed using a three-way analysis of variance (ANOVA), considering the following factors: time, N rate, and fertilizer. Winter wheat plant biomass and productivity changes under fertilization effects were analyzed with one-way ANOVA. Significant differences in the data were assessed using Tukey’s studentized range test at *p* < 0.05, with means labelled with the same letter not considered significantly different. The standard error (SE) of the mean was used to indicate the variability of the control treatment data.

## 5. Conclusions

The types of fertilizers, rates, and years (meteorological conditions) had an impact on the mineral nitrogen (N) content in the soil and on plant N nutrition. Under favourable conditions (with sufficient autumn rainfall and steady precipitation during vegetation), mineral N increased throughout the wheat growing season, peaking after harvest (120 and 120 + 50 kg ha^−1^ nitrogen rates). Under less favourable conditions (marked by alternating dry and wet periods), soil mineral N increased only during the intensive wheat growth period. In autumn–winter, soil mineral N decreased by 2.8–3.4 times compared to post-harvest levels. No N differences were observed between fertilizers and N rates when the reseeded wheat vegetation resumed.

During the intensive growth period, wheat accumulated 56.4 and 75.1% (favourable and less favourable years) of the total biomass N at maturity. Fertilizer N use efficiency (NUE) was highest with lower N rates in favourable years, while increasing rates led to lower efficiency. Similar trends were observed as cereals matured. In less favourable years, NUE was low and not dependent on fertilizer rates.

Liquid organic fertilizers—anaerobic digestate (AD) and pig slurry (PS)—were the most efficient in favourable years, while ammonium nitrate was more effective in less favourable years. The effectiveness of anaerobic digestate depends on its chemical composition. The highest N input per tonne of grain was found with the highest and medium N rates (in less favourable years). Fertilization increased total energy in grains by 43.4–48.0%, with liquid organic fertilizers increasing energy content by 49.2% (PS) and 44.1% (AD).

## Figures and Tables

**Table 1:** Influence of sampling time and fertilizer interactions on the variation in mineral N in soil (0–60 cm, mg kg^−1^) during two years of waxy winter wheat (*Triticum aestivum* L.) cultivation.

Treatments	Sampling Periods					
	2020			2021		
	1AV	1IG	1AH	2AV	2IG	2AH
N0	5.18 ± 0.08	9.46 ± 0.61	13.26 ± 0.43	4.11 ± 0.32	4.68 ± 0.63	2.38 ± 0.28
Time × N rates						
N60	5.13 cd	11.33 f	12.73 g	4.57 bc	6.14 de	3.22 a
N120	5.13 cd	11.79 e	15.61 h	4.65 bc	6.73 e	3.50 ab
N120 + 50	5.13 cd	11.49 fg	15.56 h	4.53 c	6.81 e	6.47 e
Time × fertilizer						
AN	5.13 abc	10.37 d	14.16 efg	4.26 a	7.65 d	4.53 a
PS	5.13 abc	10.93 d	14.54 efg	4.84 abc	5.87 bc	4.17 a
AD	5.13 abc	13.61 e	15.20 g	4.65 ab	6.15 c	4.49 a
Mean	5.13	11.64	14.63	4.58	6.56	4.40

Soil collection time during winter wheat growth: AV—at the beginning of spring vegetation; IG—during intensive growth (~1.5 months after AV and N fertilization); AH—after harvest. Nitrogen (N) rates: N0—unfertilized; N60—60 kg N·ha^−1^; N120—120 kg N·ha^−1^; N120 + 50—120 + 50 kg N·ha^−1^. Fertilizers: AN—ammonium nitrate; PS—pig slurry; AD—anaerobic digestate. The standard error of the mean is used to represent error values. Means with different letters within individual interactions are significantly different at the *p* < 0.05.

**Table 2:** Changes in soil mineral N concentration (0–60 cm, mg kg^−1^) during vegetation and autumn – non-vegetation periods of waxy winter wheat (*Triticum aestivum* L.).

Treatments	2020 Vegetation Period		Autumn and Non-Vegetation Period (2AV–1AH)	2021 Vegetation Period	
	The First Half (1IG–1AV)	The Second Half (1AH–1IG)		The First Half (2IG–2AV)	The Second Half (2AH–2IG)
N0	4.28 ± 0.54	3.80 ± 0.56	−9.15 ± 0.38	0.57 ± 0.37	−2.29 ± 0.47
Time × N rates					
N60	6.20 fg	1.41 cd	−8.16 ab	1.57 cd	−2.92 b
N120	6.67 g	3.82 e	−10.96 a	2.07 de	−3.22 b
N120 + 50	6.67 fg	3.77 e	−11.03 a	2.28 de	−0.34 c
Time × fertilizer					
AN	5.24 ef	3.79 ef	−9.90 a	3.40 de	−3.12 b
PS	5.81 f	3.61 e	−9.70 a	1.03 c	−1.70 b
AD	8.49 g	1.59 cd	−10.55 a	1.50 cd	−1.66 b
Mean	6.52	3.00	−10.05	1.98	−2.16

Soil collection time during winter wheat growth: AV—at the beginning of spring vegetation; IG—during intensive growth (~1.5 months after AV and N fertilization); AH—after harvest. Nitrogen (N) rates: N0—unfertilized; N60—60 kg N·ha^−1^; N120—120 kg N·ha^−1^; N120 + 50—120 + 50 kg N·ha^−1^. Fertilizers: AN—ammonium nitrate; PS—pig slurry; AD—anaerobic digestate. The standard error of the mean is used to represent error values. Means with different letters within individual interactions are significantly different at the *p* < 0.05.

**Table 3:** Above-ground biomass and its dry matter (DM) accumulation of waxy winter wheat (*Triticum aestivum* L.) at the intensive growth stage (BBCH 35, 1IG, and 2IG).

Treatments	2020				2021			
	DM		Above-Ground Mass		DM		Above-Ground Mass	
	g kg^−1^	Change * g kg^−1^	kg ha^−1^ DM	Change * kg ha^−1^	g kg^−1^	Change * g kg^−1^	kg ha^−1^ DM	Change * kg ha^−1^
N0	280.77 c	0.00	3363.6 a	0.0	229.5 bcd	0.00	1553.1 a	0.0
AN60	265.93 abc	−14.83 abc	3690.8 abc	327.2 ab	217.2 abcd	−12.27 ab	1819.5 abc	266.4 abc
PS60	268.63 abc	−12.13 abc	3627.8 abc	264.2 ab	230.8 d	1.27 d	1783.8 abc	230.6 a
AD60	257.67 a	−23.10 a	3782.8 c	419.2 ab	227.9 bcd	−1.63 bcd	1763.8 ab	210.7 a
AN120	278.83 bc	−1.93 c	3781.9 bc	418.3 ab	210.4 a	−19.07 a	2136.9 e	583.8 c
PS120	253.90 a	−26.87 a	3866.7 c	503.1 b	216.6 ab	−12.87 ab	1876.0 bcde	322.8 abc
AD120	262.90 abc	−17.87 abc	3839.5 bc	475.9 ab	226.5 bcd	−2.97 bcd	2064.8 cde	511.7 abc
Mean	266.9	−16.1	3707.6	401.3	222.7	−7.92	1856.8	303.7

Fertilizers: AN—ammonium nitrate, PS—pig slurry, AD—anaerobic digestate. Nitrogen (N) rates: N0—unfertilized; N60—60 kg N·ha^−1^; 120—120 kg N·ha^−1^. Means with different letters within individual parameter (absolute values and their respective change) are significantly different at the *p* < 0.05 level in separate years. * Variation in dry matter (DM) and above-ground mass change calculated from the control plot.

**Table 4:** Nitrogen accumulation and use efficiency in waxy winter wheat (*Triticum aestivum* L.) above-ground biomass at the intensive growth stage (BBCH 35, 1IG and 2IG).

Treatments	2020			2021		
	N Concentration, g kg^−1^ DM	N Accumulated in Biomass, kg ha^−1^	NUE, %	N Concentration, g kg^−1^ DM	N Accumulated in Biomass, kg ha^−1^	NUE, %
N0	13.66 a	43.6 a	-	21.8 a	33.9 a	-
AN60	16.39 bcd	60.5 bcd	28.1 bcd	24.89 bcd	45.2 b	18.8 ab
PS60	15.66 ab	56.9 b	22.2 abcd	22.94 ab	41.2 ab	11.9 ab
AD60	17.40 bcd	66.0 bcd	37.3 d	23.45 ab	41.5 ab	12.7 ab
AN120	15.94 bcd	60.2 b	13.8 a	26.98 d	57.8 d	19.9 b
PS120	18.11 d	70.0 d	22.0 ab	24.68 abcd	46.4 b	10.4 ab
AD120	16.85 bcd	64.7 bcd	17.6 ab	23.00 ab	47.5 bcd	11.4 ab
Mean	16.29	60.3	23.5	23.96	44.7	14.2

Fertilizers: AN—ammonium nitrate, PS—pig slurry, AD—anaerobic digestate. Nitrogen (N) rates: N0—unfertilized; N60—60 kg N·ha^−1^; N120—120 kg N·ha^−1^. NUE—N use efficiency. Means with different letters within individual parameter are significantly different at the *p* < 0.05 level in separate years.

**Table 5:** Nitrogen accumulation and use efficiency in waxy winter wheat (*Triticum aestivum* L.) yield (BBCH 87).

Treatments	2020			2021		
	N Concentration in Grain, g kg^−1^	N Accumulated in Grain–Straw Biomass, kg ha^−1^	NUE, %	N Concentration in Grain, g kg^−1^	N Accumulated in Grain–Straw Biomass, kg ha^−1^	NUE, %
N0	16.58 a	67.15 a		21.75 a	39.00 a	
AN60	16.65 ab	96.82 abcd	49.5 abc	23.17 ab	55.65 bc	27.8 ab
PS60	17.67 ab	108.80 bcd	69.4 bc	24.00 bcd	57.14 bcde	30.2 b
AD60	18.64 ab	112.09 bcd	74.9 c	24.11 bcd	48.03 ab	15.0 ab
AN120	17.91 ab	89.95 ab	19.0 a	24.35 bcd	68.96 cde	25.0 ab
PS120	19.23 bcde	118.87 bcd	43.1 abc	23.54 b	61.44 bcde	18.7 ab
AD120	17.63 ab	101.11 abcd	28.3 abc	23.33 b	62.15 bcde	19.3 ab
AN120 + 50	21.56 cde	122.01 bcd	32.3 abc	24.60 bcd	70.56 e	18.6 ab
PS120 + 50	21.96 e	135.54 d	40.2 abc	25.65 d	65.86 cde	15.8 ab
AD120 + 50	18.86 abc	116.23 bcd	28.9 abc	25.48 cd	66.87 cde	16.4 ab
Mean	18.78	106.86	42.8	24.00	59.56	20.8

Fertilizers: AN—ammonium nitrate, PS—pig slurry, AD—anaerobic digestate. Nitrogen (N) rates: N0—unfertilized; N60—60 kg N·ha^−1^; N120—120 kg N·ha^−1^; N120 + 50—120 + 50 kg N·ha^−1^. The additional N fertilization (+50) was always applied as ammonium nitrate. NUE—N use efficiency. Means with different letters within individual parameter are significantly different at the *p* < 0.05 level in separate years.

**Table 6:** The influence of fertilizers and nitrogen rates on the gross energy (GE) accumulated in the grain yield of waxy winter wheat (*Triticum aestivum* L.).

Treatments	Gross Energy GJ ha^−1^			Gross Energy index
	2020	2021	Cumulative	Relative Units
N0	59.2 a	28.0 a	87.2 a	100.0
AN60	88.2 bc	37.4 bcd	125.6 bc	144.0
PS60	90.2 bc	38.3 bcd	128.4 bc	147.2
AD60	89.3 bc	32.0 ab	121.3 bc	139.1
AN120	74.2 abc	44.3 d	118.5 bc	135.9
PS120	90.7 c	40.8 cd	131.5 c	150.8
AD120	86.8 bc	39.7 bcd	126.5 bc	145.1
AN120 + 50	84.0 bc	43.5 cd	127.5 bc	146.2
PS120 + 50	90.2 bc	40.3 bcd	130.5 bc	149.7
AD120 + 50	88.4 bc	40.7 bcd	129.1 bc	148.1
Mean	84.1	38.5	122.6	140.6

Fertilizers: AN—ammonium nitrate, PS—pig slurry, AD—anaerobic digestate. Nitrogen (N) rates: N0—unfertilized; N60—60 kg N·ha^−1^; N120—120 kg N·ha^−1^; N120 + 50—120 + 50 kg N·ha^−1^. The additional N fertilization (+50) was always applied as ammonium nitrate Data with different letters within individual interactions are significantly different at the *p* < 0.05 level.

**Table 7:** Fertilization of amylopectin starch-synthesizing waxy winter wheat (*Triticum aestivum* L.).

Treatments Abbreviation	Main Fertilization			Additional Fertilization		
	Fertilizer Name	Fertilization Time	N Rate kg ha^−1^	Fertilizer Name	Fertilization Time	N Rate kg ha^−1^
N0	Unfertilized (Control)	BBCH 25	0			
AN60	Ammonium nitrate	BBCH 25	60			
PS60	Pig slurry	BBCH 25	60			
AD60	Anaerobic digestate	BBCH 25	60			
AN120	Ammonium nitrate	BBCH 25	120			
PS120	Pig slurry	BBCH 25	120			
AD120	Anaerobic digestate	BBCH 25	120			
AN120 + 50	Ammonium nitrate	BBCH 25	120	Ammonium nitrate	BBCH 35	50
PS120 + 50	Pig slurry	BBCH 25	120	Ammonium nitrate	BBCH 35	50
AD120 + 50	Anaerobic digestate	BBCH 25	120	Ammonium nitrate	BBCH 35	50

N—nitrogen; BBCH—plant phenology classification system used to describe growth stages.

**Table 8:** The chemical composition of liquid organic fertilizers.

Year	pH	DM	OM	C_org_	N_tot_	N-NH_4_	N-NO_3_	MHS	MHA	MFA	C/N
g kg^−1^											
Pig slurry (PS)											
2020	7.65	31.6	24.1	7.41	2.36	1.65	0.018	3.40	1.18	2.22	3.14
2021	6.86	40.4	31.4	12.87	4.74	2.94	0.012	7.66	1.78	5.88	2.72
Anaerobic digestate (AD)											
2020	7.72	27.5	17.2	10.44	2.76	1.66	0.009	4.18	1.78	2.40	3.78
2021	7.77	14.0	10.1	4.46	2.38	1.79	0.017	3.12	1.37	1.75	1.87

DM—dry matter; OM—organic matter; C_org_—organic carbon, N_tot_—total nitrogen; N-NH_4_—ammonium nitrogen; N-NO_3_—nitrate nitrogen; MHS—mobile humic substances; MHA—mobile humic acids; MFA—mobile fulvic acids; C/N—carbon-to-nitrogen ratio.

**Table 9:** Meteorological conditions: monthly average rainfall and temperature according to the meteorological site of the LAMMC Joniškėlis experimental station.

Month	Temperature, °C			Precipitation, mm		
	2019–2020	2020–2021	SCN (1991–2020)	2019–2020	2020–2021	SCN (1991–2020)
September	13.8	16.2	12.0	57.0	17.7	57.9
October	10.5	12.2	6.3	34.1	60.6	45.5
November	5.0	5.2	1.4	45.1	65.3	42.7
December	2.6	0.8	−3.0	31.7	23.6	39.0
January	2.8	−5.7	−4.7	26.1	51.6	31.6
February	2.8	−9.6	−5.0	33.2	9.0	26.9
March	3.7	−2.0	−0.6	26.0	35.0	26.6
April	6.4	7.3	6.6	14.9	11.4	33.7
May	10.5	13.7	12.3	44.8	137.2	45.6
June	20.2	23.5	15.5	104.2	69.7	60.0
July	19.1	25.2	17.2	113.7	32.9	72.8
August	22.1	17.6	17.0	26.7	176.0	63.5
Mean/sum	9.96	13.52	11.04	557.5	657.1	545.8

SCN—standard climatic norm.

## Data Availability

The original contributions presented in the study are included in the article, further inquiries can be directed to the corresponding author.
